# Efficacy and Safety of Fexuprazan‐Based Modified High‐Dose Dual Therapy for *Helicobacter pylori* Eradication: A Randomized Clinical Trial

**DOI:** 10.1111/hel.70146

**Published:** 2026-06-08

**Authors:** Ji Yong Ahn, Ki‐Nam Shim, Jung‐Ho Park, Sang Gyun Kim, Jeong Hwan Kim, Jeong Seop Moon, Young Hoon Youn, Jae J Kim

**Affiliations:** ^1^ Division of Gastroenterology, Department of Internal Medicine University of Ulsan College of Medicine, Asan Medical Center Seoul Korea; ^2^ Department of Internal Medicine Ewha Womans University College of Medicine Seoul Korea; ^3^ Department of Medicine Sungkyunkwan University School of Medicine, Kangbuk Samsung Hospital Seoul South Korea; ^4^ Department of Internal Medicine and Liver Research Institute Seoul National University College of Medicine Seoul Korea; ^5^ Department of Internal Medicine Konkuk University School of Medicine Seoul Korea; ^6^ Department of Internal Medicine Sanggye Paik Hospital, Inje University College of Medicine Seoul Korea; ^7^ Division of Gastroenterology, Department of Internal Medicine Gangnam Severance Hospital, Yonsei University College of Medicine Seoul Korea; ^8^ Department of Gastroenterology Ujeongbu Eulji Medical Center Gyenggi‐do Korea

**Keywords:** bismuth, eradication, *Helicobacter pylori*, potassium‐competitive acid blocker

## Abstract

**Background/Aims:**

The eradication efficacy of proton pump inhibitor (PPI)–based standard triple therapy (STT) for *Helicobacter pylori* infection has declined in Korea, largely because of increasing clarithromycin resistance. High‐dose dual therapy (HDDT) using potent acid suppression represents a promising clarithromycin‐sparing strategy. This study evaluated the efficacy and safety of fexuprazan‐based modified HDDT (m‐HDDT) including bismuth, compared with conventional PPI‐based STT as first‐line eradication therapy.

**Methods:**

This prospective, multicenter, randomized, open‐label, non‐inferiority trial was conducted at eight tertiary hospitals in Korea. Treatment‐naïve adults with confirmed *H. pylori* infection were randomized to receive either m‐HDDT (fexuprazan 40 mg twice daily, amoxicillin 1000 mg three times daily, and bismuth subcitrate potassium 300 mg three times daily) or STT (lansoprazole 30 mg, amoxicillin 1000 mg, and clarithromycin 500 mg, all twice daily) for 14 days. Eradication was assessed by urea breath test 4–8 weeks after therapy. The primary endpoint was the eradication rate in the full analysis set (FAS), with a prespecified non‐inferiority margin of −10%. Safety and compliance were evaluated as secondary outcomes.

**Results:**

In total, 196 patients were included in the FAS (m‐HDDT, *n* = 96; STT, *n* = 100). *Helicobacter pylori* eradication was achieved in 81.3% of patients in the m‐HDDT group and 79.0% in the STT group, demonstrating non‐inferiority of m‐HDDT (one‐sided Wald test, *p* = 0.0158). Per‐protocol analysis yielded consistent results (*p* = 0.0215). Drug compliance was high in both groups, with mean compliance rates of 97.0% in the m‐HDDT group and 99.0% in the STT group; more than 95% of patients in each group achieved ≥ 80% compliance. The incidence of treatment‐emergent adverse events was comparable between groups (16.7% vs. 14.0%); most events consisted of mild gastrointestinal symptoms. No serious adverse events or treatment discontinuations due to adverse events were observed in either group.

**Conclusions:**

Fexuprazan‐based m‐HDDT with bismuth was non‐inferior to PPI‐based STT for *H. pylori* eradication and demonstrated comparable safety and excellent compliance. This clarithromycin‐sparing regimen can be considered an alternative treatment option in regions with high macrolide resistance.

## 1 Introduction

*Helicobacter pylori* infection represents a major global health burden and is classified as a class I carcinogen by the World Health Organization, given that it is the most important risk factor for peptic ulcer disease and gastric cancer. The prevalence of *H. pylori* infection in Korea has historically exceeded 40%; although the rate has declined in recent years, this infection continues to pose a substantial clinical challenge [1, 2]. For more than two decades, standard triple therapy (STT)—typically consisting of a proton pump inhibitor (PPI), clarithromycin, and amoxicillin—has been used worldwide as first‐line treatment. However, eradication rates with STT have declined to below 80% in Western countries and are even lower in Korea, where recent data indicate rates of only 60%–70% after 1 week of therapy [3]. The principal cause of this decline is the high prevalence of clarithromycin resistance in Korea, estimated at approximately 20%–30% [4, 5].

To address these limitations of STT and reduce unnecessary antibiotic exposure, regimens based on high‐dose dual therapy (HDDT) have increasingly been introduced. HDDT is supported by well‐established pharmacologic principles describing the interaction among acid suppression, bacterial physiology, and amoxicillin activity. *Helicobacter pylori* is most susceptible to amoxicillin when intragastric pH is maintained at ≥ 6, a condition under which the organism shifts from a dormant to a replicative state and becomes vulnerable to cell wall–active antibiotics [6]. Potent and sustained acid inhibition also improves the stability, intragastric concentration, and bioavailability of amoxicillin. Additionally, amoxicillin exhibits time‐dependent killing, has a short plasma half‐life of approximately 1 h, and lacks a clinically meaningful post‐antibiotic effect, indicating that higher or more frequent dosing increases the duration above the minimal inhibitory concentration and enhances bactericidal activity [6]. In a large phase 3 randomized trial conducted in the United States and Europe, both vonoprazan‐based triple therapy and dual therapy achieved significantly higher eradication rates than PPI‐based triple therapy, particularly among infections caused by clarithromycin‐resistant strains [7].

Bismuth has long been recognized as a valuable adjunctive agent in *H. pylori* eradication therapy because it enhances treatment efficacy through multiple complementary mechanisms. Bismuth exerts direct antimicrobial activity by disrupting bacterial cell walls, inhibiting urease activity, and impairing adhesion of *H. pylori* to the gastric epithelium [8]. Furthermore, bismuth forms a protective mucosal coating that stabilizes the gastric environment and improves the local activity of coadministered antibiotics [9, 10]. Bismuth‐containing regimens have consistently demonstrated high eradication rates regardless of clarithromycin resistance; their efficacy is largely unaffected by CYP2C19 polymorphisms or regional resistance patterns [11]. These properties make bismuth an effective component for reinforcing non–clarithromycin‐based strategies such as HDDT, in which it may enhance bacterial clearance without increasing antibiotic burden.

When a more potent acid suppressor, such as a potassium‐competitive acid blocker (P‐CAB), is used instead of a conventional PPI, the eradication rate of STT can be improved; however, HDDT efficacy may increase even further under the enhanced acid suppression achieved with P‐CAB. Moreover, the addition of bismuth to HDDT is expected to further improve eradication efficacy without requiring additional antibiotics, providing a strategically advantageous approach in regions with rising macrolide resistance. Based on this rationale, the present study aimed to compare the eradication efficacy and safety of a modified HDDT regimen incorporating a P‐CAB and bismuth with those of STT.

## 2 Methods

### 2.1 Study Design

This prospective, multicenter, randomized, open‐label, active‐controlled, investigator‐initiated clinical trial was conducted at eight tertiary hospitals in Korea. The study was performed in accordance with the Declaration of Helsinki and the Korean Good Clinical Practice guidelines. The study protocol (version 1.1, September 26, 2023) was approved by the institutional review board of Asan Medical Center (No. 2023‐0732), and all participants provided written informed consent. The trial was registered with the Korean Disease Control and Prevention Agency Clinical Research Information Service (KCT0008592; available at: https://cris.nih.go.kr/cris/search/detailSearch.do?search_lang=E&focus=reset_12&search_page=L&pageSize=10&page=undefined&seq=32370&status=5&seq_group=25093). This study was conducted and reported in accordance with the Consolidated Standards of Reporting Trials (CONSORT) guidelines.

### 2.2 Participants

Eligible participants were adults aged 20–79 years who were undergoing upper gastrointestinal endoscopy for dyspeptic symptoms, evaluation of suspected peptic ulcer disease or gastric neoplasia, or routine gastric cancer screening. *Helicobacter pylori* infection was required to be confirmed by urea breath test, rapid urease test, histology, or culture performed within 4–8 weeks before enrollment. Only individuals capable of understanding the study procedures and voluntarily providing written informed consent were included. Patients were excluded if they had received prior *H. pylori* eradication therapy; had known hypersensitivity to penicillins, macrolides, bismuth compounds, PPIs, or P‐CAB agents; or required medications that represented contraindications for the study drugs. Use of PPIs, H2‐receptor antagonists, or P‐CABs within 2 weeks before randomization, as well as use of antibiotics or bismuth compounds within 4 weeks, was not permitted. Patients with clinically significant hepatic dysfunction (aspartate aminotransferase [AST], alanine transaminase [ALT], alkaline phosphatase [ALP], gamma‐glutamyl transferase [γ‐GT], or total bilirubin ≥ 2× the upper limit of normal), impaired renal function (blood urea nitrogen [BUN] or creatinine ≥ 1.5× the upper limit of normal), uncontrolled diabetes mellitus or hypertension, or active gastrointestinal bleeding were excluded. Additional exclusion criteria were a history of gastric or esophageal surgery; gastrointestinal malignancy within 5 years (except for endoscopically treated early gastric cancer or adenoma); genetic lactose metabolism disorders; alcohol dependence; pregnancy or breastfeeding; or refusal to agree to adequate contraception during the study period. Investigators also excluded individuals considered unsuitable for trial participation due to safety concerns or anticipated non‐compliance.

### 2.3 Randomization and Interventions

Participants who satisfied all eligibility criteria were randomized at a 1:1 ratio to receive either fexuprazan‐based modified HDDT or PPI‐based STT. Randomization was performed at the baseline visit (Day 0) after confirmation of *H. pylori* infection and final verification of inclusion and exclusion criteria. The trial followed an open‐label design consistent with investigator‐initiated, practice‐based clinical studies; no additional stratification factors were utilized. Study medications were dispensed by certified clinical research pharmacists at each participating institution, and treatment compliance was assessed during follow‐up visits.

Participants assigned to the fexuprazan‐based modified HDDT (m‐HDDT group) received fexuprazan 40 mg twice daily, amoxicillin 1000 mg three times daily, and bismuth subcitrate potassium 300 mg three times daily for 14 days. Participants randomized to the PPI‐based STT (STT group) received lansoprazole 30 mg twice daily, amoxicillin 1000 mg twice daily, and clarithromycin 500 mg twice daily for 14 days. All medications were administered orally. Patients were instructed to avoid additional acid suppressants, bismuth‐containing preparations, or antibiotics from 4 weeks before randomization until completion of the test‐of‐cure assessment.

Participants were advised to maintain regular dosing intervals throughout the 14‐day treatment period. Compliance was evaluated by pill counts, patient interviews, and review of medication diaries at the follow‐up visit. Secondary therapy for persistent infection after trial participation was provided with bismuth‐based quadruple therapy (bismuth, metronidazole, tetracycline, and a PPI for 2 weeks) (Figure S1). Post‐randomization exclusions were limited to participants who did not receive any study medication, withdrew consent before treatment, or lacked any post‐baseline efficacy assessment. These criteria were applied equally to both treatment groups.

### 2.4 Assessments and Follow‐Up

Baseline demographic characteristics, medical history, gastrointestinal symptoms, vital signs, laboratory findings, and pregnancy testing (when applicable) were obtained at the screening visit. Assessments of resistance to amoxicillin and clarithromycin were performed only at institutions where antibiotic susceptibility testing was available.

Randomized participants returned on Day 0 for treatment initiation. At this visit, study medications were dispensed; instructions regarding dosing schedules and restrictions on concomitant medications were reviewed. Safety laboratory tests (e.g., liver and renal function panels) and pregnancy testing were repeated when indicated. During the 14‐day treatment period, participants were instructed to maintain consistent dosing intervals and to record medication intake and any adverse events in a diary.

Follow‐up evaluation was conducted 4–8 weeks after treatment completion. At this visit, participants underwent a urea breath test to assess *H. pylori* eradication. Safety assessments—including adverse event monitoring, physical examination, vital signs, and laboratory testing—were repeated. Compliance was evaluated through pill counts, documented dosing records, and structured interviews. Participants with persistent *H. pylori* infection were referred for rescue therapy in accordance with institutional clinical practice guidelines (Figure S1).

### 2.5 Outcome Measures

The primary outcome was the *H. pylori* eradication rate assessed 4–8 weeks after treatment completion and confirmed by urea breath test. Eradication was defined as a negative test‐of‐cure result obtained after discontinuation of acid suppressants (≥ 2 weeks) and antibiotics or bismuth‐containing compounds (≥ 4 weeks) prior to testing. Missing or invalid post‐treatment breath test results were classified as treatment failures, in accordance with standard intention‐to‐treat principles.

Secondary outcomes included medication compliance, evaluated by pill counts and patient diaries, and safety outcomes, including the incidence of treatment‐emergent adverse events, adverse drug reactions, serious adverse events, and clinically significant abnormalities in laboratory parameters or vital signs. All adverse events were coded and assessed according to the definitions and reporting standards specified in Korean Good Clinical Practice guidelines. Additional secondary endpoints included eradication rates stratified by baseline antimicrobial susceptibility, specifically clarithromycin and amoxicillin resistance profiles determined by culture‐based testing at institutions where antibiotic susceptibility testing was available.

### 2.6 Statistical Analysis

The primary efficacy analysis assessed the non‐inferiority of fexuprazan‐based modified HDDT relative to PPI‐based STT with respect to the proportion of patients achieving *H. pylori* eradication 4–8 weeks after treatment completion. The full analysis set (FAS), corresponding to a modified intention‐to‐treat (mITT) population, comprised all randomized participants who received at least one dose of study medication and had at least one post‐baseline efficacy assessment. The per‐protocol (PP) population included participants who completed at least 75% of the prescribed study medication, complied with all major protocol requirements, and underwent a valid test‐of‐cure assessment within the protocol‐defined window. Missing post‐treatment urea breath test results were treated as eradication failures, consistent with conservative analytical approaches commonly applied in eradication trials.

### 2.7 Sample Size Calculation

Sample size estimation was based on an expected eradication rate of approximately 80% for both treatment regimens, consistent with contemporary Korean eradication outcomes and previously published studies evaluating high‐dose dual therapy strategies. Using a non‐inferiority margin of −10%, which is consistent with margins adopted in prior *H. pylori* eradication trials involving P‐CAB–based therapy, the protocol specified that a minimum of 81 evaluable participants per group would provide sufficient statistical power to demonstrate non‐inferiority between the two regimens. Considering an anticipated dropout rate of approximately 10%, the target enrollment was increased to 90 participants per group, resulting in a planned total sample size of 180 patients. Because this was a multicenter study, a small degree of over‐enrollment occurred during the active recruitment period before final enrollment closure, reflecting routine recruitment dynamics across participating centers. This did not alter the assumptions of the original sample size calculation.

### 2.8 Efficacy Analyses

For the primary endpoint, eradication rates were compared between groups using a one‐sided Wald test; two‐sided 95% confidence intervals (CIs) were calculated for the between‐group difference. Non‐inferiority of the experimental regimen was concluded if the lower bound of the 95% CI exceeded the prespecified margin of −10%. Secondary analyses evaluated eradication rates stratified by baseline antimicrobial susceptibility, including clarithromycin‐resistant, amoxicillin‐resistant, and susceptible strains. Group comparisons were performed using the chi‐squared test or Fisher's exact test, as appropriate. The same analytical methods were applied to both the FAS and PP populations.

### 2.9 Safety Analyses

Safety outcomes, including treatment‐emergent adverse events, adverse drug reactions, serious adverse events, and clinically significant laboratory abnormalities, were assessed in all participants who received at least one dose of study medication (safety analysis set). Descriptive statistics were used to summarize adverse event incidence; between‐group comparisons were conducted using chi‐square or Fisher's exact tests when indicated. Continuous laboratory variables were summarized using the mean, standard deviation, median, and range, whereas categorical variables were presented as frequencies and percentages. All analyses were performed in accordance with Korean Good Clinical Practice guidelines using validated statistical software with SAS 9.4 (SAS Institute Inc., Cary, NC, USA). Figures were created using R version 4.4.2 (R Foundation for Statistical Computing, Vienna, Austria). The study protocol and statistical analysis plan are provided as Figures S2 and S3.

## 3 Results

### 3.1 Study Population

**FIGURE 1:**
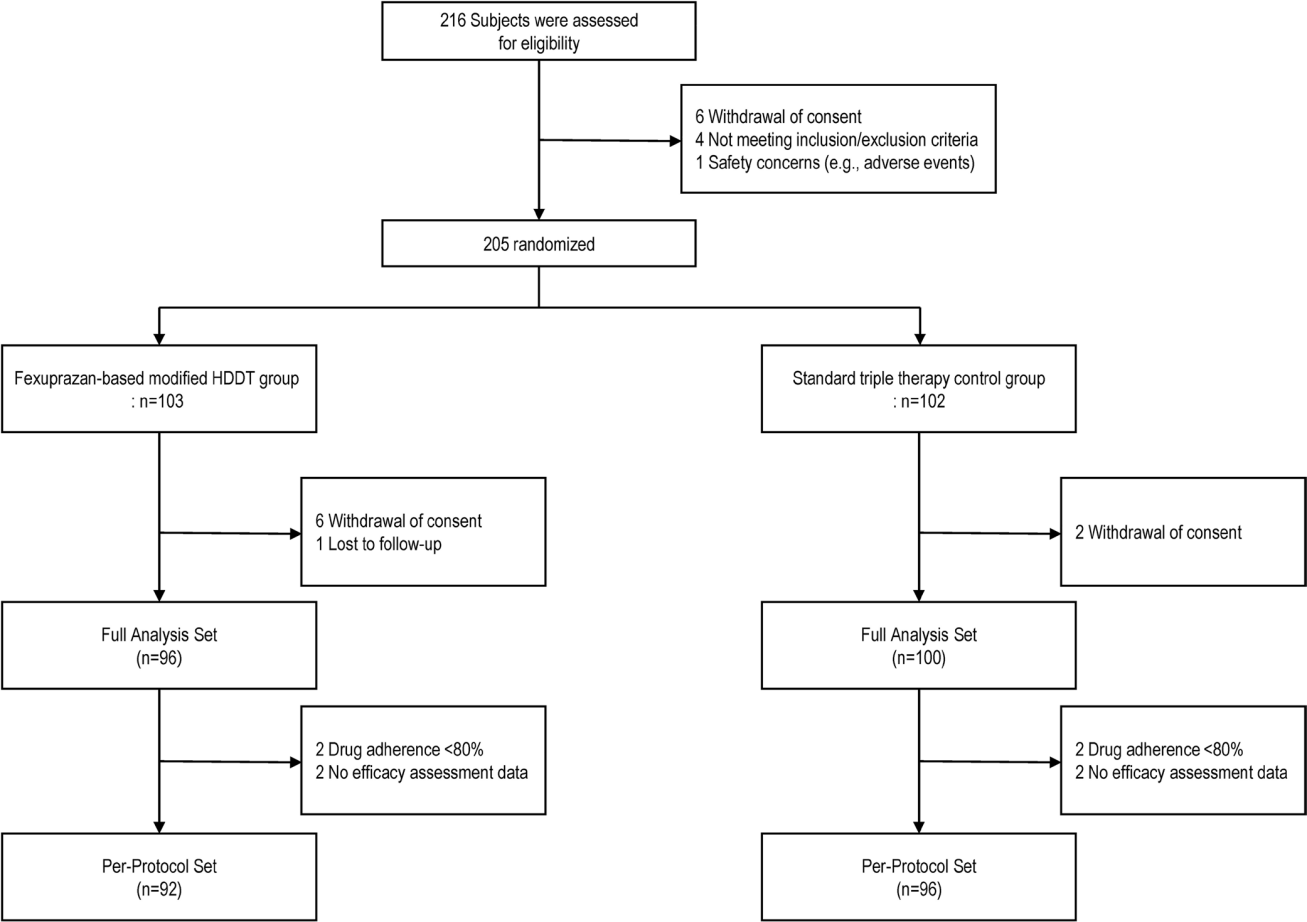
Flow chart of this study m‐HDDT group, fexuprazan‐based modified high‐dose dual therapy group; STT group, proton pump inhibitor‐based standard triple therapy group.

Of the 216 individuals screened, 205 were enrolled. After exclusion of participants who did not receive study medication, withdrew consent, or were lost to follow‐up, 196 participants comprised the FAS: 96 in the m‐HDDT group and 100 in the STT group. The PP population included 92 and 96 participants, respectively (Figure 1). Baseline demographic characteristics were well balanced between the two groups (Table 1). Mean ages were 59.7 years in the m‐HDDT group and 60.7 years in the STT group; the proportions of male participants were comparable (53.4% vs. 55.6%). Body mass index, smoking status, alcohol consumption patterns, and gastrointestinal symptoms did not significantly differ between groups.

**TABLE 1:** Baseline characteristics of enrolled patients.

Baseline factors	m‐HDDT group (*n* = 96)	STT group (*n* = 100)	*p*
Age, years, mean (SD)	59.7 ± 10.7	60.7 ± 10.6	0.4517
Sex			0.6429
Male	55 (57.3%)	54 (54.0%)	
Female	41 (42.7%)	46 (46.0%)	
BMI, kg/m^2^, mean (SD)	24.2 ± 3.0	23.8 ± 3.0	0.4002
Smoking status			0.5005
Non‐smoker	65 (67.8%)	64 (64.0%)	
Smoker	16 (16.7%)	14 (14.0%)	
Former smoker	15 (15.5%)	22 (22.0%)	
Alcohol consumption			0.4662
Non‐drinker	37 (38.5%)	39 (39.0%)	
Drinker	49 (51.0%)	45 (45.0%)	
Former drinker	10 (10.5%)	16 (16.0%)	
Gastrointestinal symptoms			
Abdominal pain	10 (10.4%)	6 (6.0%)	0.2589
Nausea/vomiting	7 (7.3%)	5 (5.0%)	0.5035
Heartburn	29 (30.2%)	36 (36.0%)	0.3893
Dyspepsia	24 (25.0%)	26 (26.0%)	0.8725

Abbreviations: BMI, body mass index; m‐HDDT group, fexuprazan‐based modified high‐dose dual therapy group; SD, standard deviation; STT group, proton pump inhibitor–‐based standard triple therapy group.

### 3.2 Eradication Success Rates in the m‐HDDT and STT Groups

**FIGURE 2:**
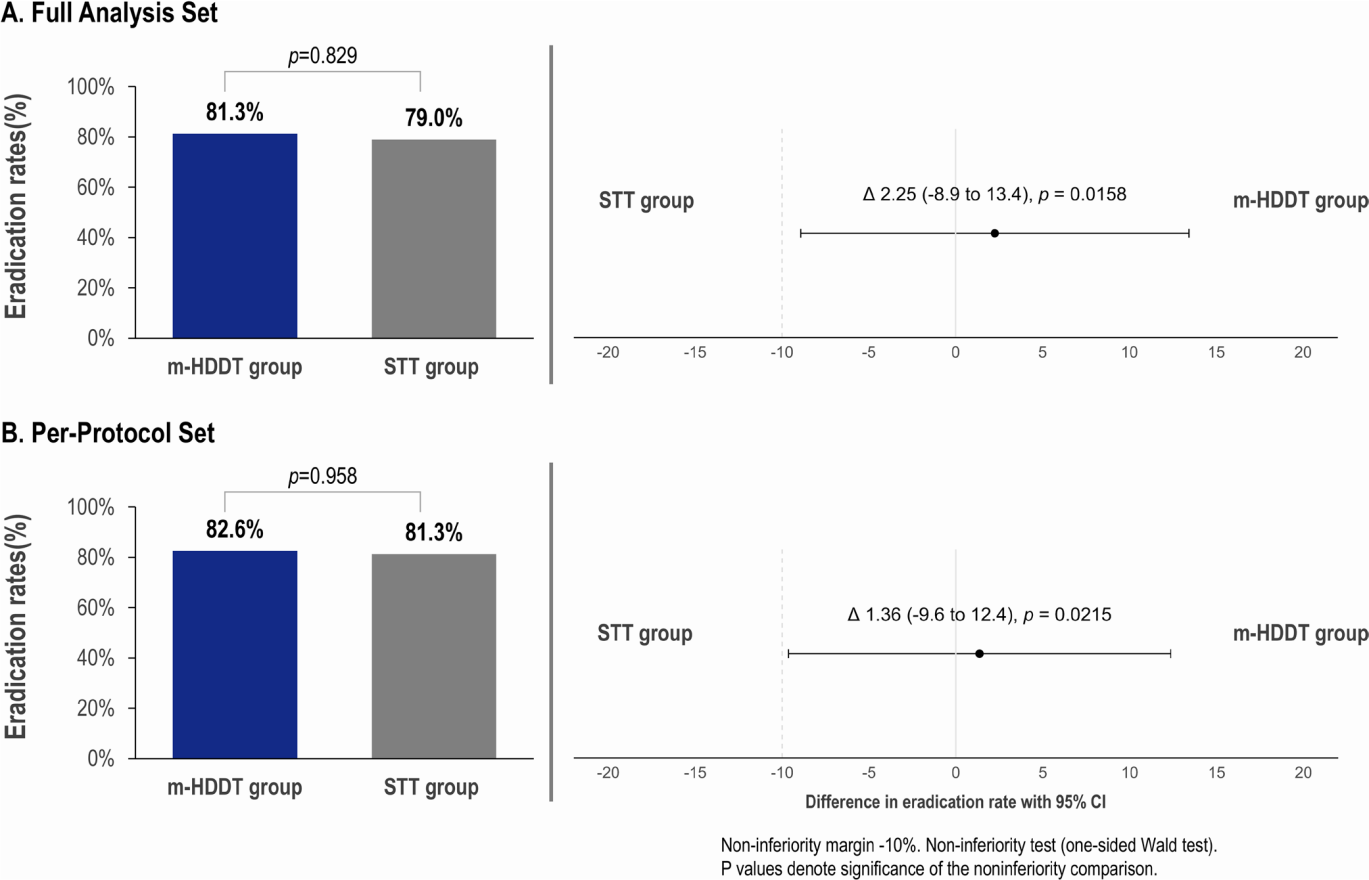
Eradication rates of the m‐HDDT group compared to the STT group in FAS and PP populations. FAS, full set analysis; PP, per protocol; m‐HDDT group, fexuprazan‐based modified high‐dose dual therapy group; STT group, proton pump inhibitor‐based standard triple therapy group; CI, confidence interval.

In the FAS population, *H. pylori* eradication was achieved in 78 of 96 patients (81.3%) in the m‐HDDT group and 79 of 100 patients (79.0%) in the STT group (difference, 2.3%; 95% CI, −8.8 to 13.4; non‐inferiority *p* = 0.0158) (Figure 2 and Table 2). Sensitivity analysis using the PP population yielded consistent results; eradication was achieved in 76 of 92 patients (82.6%) in the m‐HDDT group and 78 of 96 patients (81.3%) in the STT group (difference, 1.4%; 95% CI, −9.6 to 12.4; non‐inferiority *p* = 0.0215) (Figure 2 and Table 2).

**TABLE 2:** Eradication rates in the FAS and PP populations.

	m‐HDDT group (*n* = 96)	STT group (*n* = 100)	Risk difference, %	Difference 95% CI	*p* ^a^
FAS	81.3% (78/96)	79.0% (79/100)	2.3	−8.8 to 13.4	0.0158
PP	82.6% (76/92)	81.3% (78/96)	1.4	−9.6 to 12.4	0.0215

Abbreviations: CI, confidence interval; FAS, full analysis set; m‐HDDT group, fexuprazan‐based modified high‐dose dual therapy group; PP, per‐protocol; STT group, proton pump inhibitor–based standard triple therapy group.

^a^ The non‐inferiority margin was set at −10%. *p* values were derived from a one‐sided Wald test and indicate statistical significance for the non‐inferiority comparison rather than differences in eradication rates between groups.

Drug compliance was high in both groups; mean compliance rates were 97.0% in the m‐HDDT group and 99.0% in the STT group in the FAS analysis (Table 3). Subgroup analysis according to compliance showed no meaningful differences. Among participants with compliance ≥ 80%, eradication rates were 82.6% (76/92) in the m‐HDDT group and 79.6% (78/98) in the STT group (Table 4). In the PP analysis, eradication rates were 82.6% (76/92) in the m‐HDDT group and 81.3% (78/96) in the STT group (*p* = 0.8088).

**TABLE 3:** Drug compliance according to treatment regimen in the FAS population.

	m‐HDDT group (*n* = 96)	STT group (*n* = 100)	*p*
Mean compliance	97.0%	99.0%	0.3950
Compliance category			
< 80%	4 (4.2%)	2 (3.1%)	0.4377
≥ 80%	92 (95.8%)	98 (96.9%)	

Abbreviations: FAS, full analysis set; m‐HDDT group, fexuprazan‐based modified high‐dose dual therapy group; STT group, proton pump inhibitor–based standard triple therapy group.

**TABLE 4:** Eradication rates according to drug compliance in the FAS population.

Drug compliance	Treatment	Eradication rate	95% CI	*p*
< 80%	m‐HDDT group	2/4 (50.0%)	1.0%–99.0%	> 0.9999
	STT group	1/2 (50.0%)	0%–100%	
≥ 80%	m‐HDDT group	76/92 (82.6%)	74.9%–90.4%	0.5959
	STT group	78/98 (79.6%)	71.6%–87.6%	

Abbreviations: CI, confidence interval; m‐HDDT group, fexuprazan‐based modified high‐dose dual therapy group; STT group, proton pump inhibitor‐based standard triple therapy group.

### 3.3 Eradication Success Rates According to Antibiotic Resistance

Among participants who underwent antibiotic susceptibility testing, all 20 isolates were susceptible to amoxicillin, and no amoxicillin‐resistant strains were identified. Clarithromycin susceptibility was observed in 16 cases, whereas resistance was identified in four cases. Among amoxicillin‐susceptible patients, eradication rates were 75.0% (6/8) in the m‐HDDT group and 91.7% (11/12) in the STT group. In clarithromycin‐susceptible cases, eradication was achieved in 83.3% (5/6) of patients in the m‐HDDT group and 100% (10/10) of those in the STT group. Among clarithromycin‐resistant patients, eradication success was observed in 50.0% (1/2) of patients in each group (Table 5).

**TABLE 5:** Eradication rates according to antibiotic resistance status.

Antibiotic	Resistance status	Treatment	Eradication rate	95% CI	*p*
Amoxicillin	Resistant	m‐HDDT group	—	—	—
		STT group	—	—	—
	Susceptible	m‐HDDT group	6/8 (75.0%)	45.0%–100%	0.5368
		STT group	11/12 (91.7%)	76.0%–100%	
Clarithromycin	Resistant	m‐HDDT group	1/2 (50.0%)	0%–100%	> 0.9999
		STT group	1/2 (50.0%)	0%–100%	
	Susceptible	m‐HDDT group	5/6 (83.3%)	53.5%–100%	0.3750
		STT group	10/10 (100%)	—	
Amoxicillin and clarithromycin	Resistant	m‐HDDT group	—	—	—
		STT group	—	—	—
	Susceptible	m‐HDDT group	5/6 (83.3%)	53.5%–100%	0.3750
		STT group	10/10 (100%)	—	

Abbreviations: CI, confidence interval; m‐HDDT group, fexuprazan‐based modified high‐dose dual therapy group; STT group, proton pump inhibitor–based standard triple therapy group.

### 3.4 Overall Safety Profile

Overall incidences of treatment‐emergent adverse events (TEAEs) were 16.7% (16/96) in the m‐HDDT group and 14.0% (14/100) in the STT group, with no significant difference between groups (*p* = 0.6042). No serious TEAEs were observed in either group, and no participants discontinued therapy due to adverse events (Table 6). Additionally, no clinically meaningful abnormalities in vital signs or laboratory parameters were identified during the study period, indicating a favorable systemic safety profile for both regimens (Table S1).

**TABLE 6:** Adverse events according to treatment regimen.

	m‐HDDT group (*n* = 96)	STT group (*n* = 100)	*p*
TEAEs, *n* (%)	16 (16.7)	14 (14.0)	0.6042
Serious TEAEs, *n* (%)	0	0	
TEAEs leading to discontinuation, *n* (%)	0	0	
Most common TEAEs (≥ 2% in any group), *n* (%)			
Dyspepsia	5 (5.2)	5 (5.0%)	
Feces discolored	4 (4.2)	4 (4.0%)	
Diarrhea	3 (3.1)	4 (4.0)	
Nausea	2 (2.1)	2 (2.0)	
Vomiting	2 (2.1)	1 (1.0)	
Pollakiuria	2 (2.1)	0	
Dizziness	0	2 (2.0)	
Dysgeusia	1 (1.0)	4 (4.0)	

Abbreviations: m‐HDDT group, fexuprazan‐based modified high‐dose dual therapy group; STT group, proton pump inhibitor–based standard triple therapy group; TEAEs, treatment‐emergent adverse events.

TEAEs occurring in ≥ 2% of patients in either group were primarily gastrointestinal. The most frequently reported events included dyspepsia (5.2% vs. 5.0%), feces discolored (4.2% vs. 4.0%), diarrhea (3.1% vs. 4.0%), and dysgeusia (1.0% vs. 4.0%) in the m‐HDDT and STT groups, respectively. Other gastrointestinal symptoms, including nausea (2.1% vs. 2.0%) and vomiting (2.1% vs. 1.0%), were also reported; frequencies were similarly low in both groups. In addition, no serious TEAEs were observed in both groups, and no participants discontinued treatment because of adverse events.

Among participants who experienced TEAEs, eradication rates were 68.8% (11/16) in the m‐HDDT group and 92.9% (13/14) in the STT group (*p* = 0.1755). Among participants without TEAEs, eradication rates were 83.8% (67/80) in the m‐HDDT group and 76.7% (66/86) in the STT group (*p* = 0.2584).

### 3.5 Secondary Eradication After Treatment Failure

Among 39 patients with treatment failure, second‐line therapy with a bismuth‐based quadruple regimen was administered to 26 patients, yielding an overall eradication rate of 92.3% (24/26). Within the FAS population, eradication was achieved in 81.3% of patients in the m‐HDDT group and 79.0% of those in the STT group, corresponding to a risk difference of 2.3%. Within the PP population, eradication rates were 82.6% and 81.3%, respectively, resulting in a risk difference of 1.4%. Using the predefined non‐inferiority margin of −10%, non‐inferiority of the m‐HDDT regimen to the STT regimen was demonstrated in both the FAS and PP analyses based on the one‐sided Wald test.

## 4 Discussion

This study is the first prospective randomized clinical trial to evaluate a fexuprazan‐based HDDT regimen for *H. pylori* eradication. This multicenter trial in Korea demonstrated that fexuprazan‐based modified HDDT including bismuth was non‐inferior to conventional PPI‐based STT as first‐line therapy. In the FAS analysis, eradication was achieved in 81.3% of patients receiving fexuprazan‐based m‐HDDT and in 79.0% of those receiving PPI‐based STT; medication compliance and rates of treatment‐emergent adverse events were comparable between groups. P‐CABs provide more rapid and sustained acid suppression than conventional PPIs, offering a strong pharmacologic rationale for HDDT strategies. Bismuth may exert synergistic effects on eradication. In the context of persistently high clarithromycin resistance and declining efficacy of standard triple therapy, these findings suggest that a fexuprazan‐based m‐HDDT regimen incorporating bismuth can be effective as a clinically meaningful clarithromycin‐sparing first‐line treatment option for *H. pylori* eradication.

The rationale for m‐HDDT is based on the pharmacodynamic principle that sustained elevation of intragastric pH optimizes the time‐dependent bactericidal activity of amoxicillin. A recent randomized trial in Japan revealed that 7‐day vonoprazan plus low‐dose amoxicillin dual therapy achieved eradication rates comparable to vonoprazan‐based triple therapy (84.5% vs. 89.2%, *p* = 0.203). Subsequent randomized and non‐inferiority trials have shown that 14‐day vonoprazan–amoxicillin dual regimens achieved intention‐to‐treat eradication rates of 92.0% and demonstrated non‐inferiority to bismuth‐based quadruple therapy (88.0%) [12, 13]. Meta‐analyses suggest that HDDT using either a PPI or a P‐CAB is as effective as bismuth‐containing quadruple therapy, with fewer adverse events and similar or improved compliance, supporting the use of HDDT as a rational regimen simplification strategy [14].

In the present study, the eradication rate of m‐HDDT was approximately 81.3% in FAS population and 82.6% in PP population, which is comparable to those of STT but somewhat lower than rates reported in some previous HDDT studies. One possible explanation for this discrepancy is the evolving antimicrobial susceptibility pattern in Korea. Recent Korea‐specific MIC distribution data suggested that the tentative resistance rate to amoxicillin may be as high as 17.9% [15]. Because HDDT relies primarily on the combination of potent acid suppression and amoxicillin, reduced amoxicillin susceptibility in local strains may have attenuated the efficacy of our regimen. Although amoxicillin resistance was not confirmed in our trial cohort, as susceptibility testing was performed only in a subset of patients, this contemporary resistance profile may partly explain the relatively modest eradication rate observed in our study. In addition, pharmacodynamic considerations related to amoxicillin may also have influenced the eradication rate observed in this study. As amoxicillin exerts time‐dependent bactericidal activity, maintaining sufficient time above the minimum inhibitory concentration is critical for optimal efficacy. The three‐times‐daily dosing regimen used in our study may not have fully optimized pharmacodynamic exposure in all patients, particularly in the presence of reduced bacterial susceptibility. Although the eradication rate of HDDT in our study was somewhat lower than that reported in previous studies, within this evolving therapeutic landscape, the present trial provides the first prospective evidence that fexuprazan‐based m‐HDDT including bismuth can achieve eradication outcomes comparable to those of established PPI‐based STT under routine clinical practice conditions in Korea.

Compliance with *H. pylori* eradication therapy is a critical determinant of clinical effectiveness, particularly for regimens that require multiple daily doses and combinations of antibiotics. Previous studies comparing triple and quadruple regimens have generally demonstrated high but variable compliance, typically ranging from 85% to 95%; these findings have suggested that regimen complexity and adverse drug reactions, rather than the specific combination of agents, are the primary contributors to non‐compliance [16, 17]. More recent trials of vonoprazan–amoxicillin dual therapy have consistently shown high compliance, with at least 80% compliance observed in 98.2% of patients, and low discontinuation rates of 1.2%, reinforcing the concept that simplified regimens with potent acid suppression can be both effective and well tolerated [12]. In the present study, overall medication compliance was excellent and closely aligned with these prior reports, despite the more frequent dosing required in the m‐HDDT group. Mean compliance exceeded 95% in both groups (7.0% in the m‐HDDT group and 990% in the STT group), and the proportion of participants achieving ≥ 80% compliance was similarly high (958% and 96.9%, respectively); there was no statistically significant difference between regimens. Among patients with compliance ≥ 80%, eradication rates remained above 80% in both groups (82.6% in the m‐HDDT group and 79.6% in the STT group), indicating that the intensified amoxicillin and bismuth dosing schedule did not compromise treatment completion or overall effectiveness. These findings suggest that fexuprazan‐based m‐HDDT does not pose a unique barrier to compliance and can be implemented in routine clinical practice without substantial concern regarding reduced compliance.

Several recent studies have evaluated HDDT in the context of antibiotic susceptibility testing, providing important insights into the performance of dual therapy relative to clarithromycin‐containing regimens in resistant strains. A meta‐analysis noted that, in clarithromycin‐resistant *H. pylori* , vonoprazan–amoxicillin dual therapy achieved a pooled cure rate of 86.7%, which was significantly higher than the cure rate of vonoprazan‐based triple therapy (71.4%). In contrast, among clarithromycin‐susceptible strains, the triple regimen demonstrated superior outcomes compared with vonoprazan–amoxicillin dual therapy (83.0% vs. 71.4%) [18]. In a randomized trial involving patients with at least one prior eradication failure, high‐dose esomeprazole–amoxicillin dual therapy was non‐inferior to culture‐based susceptibility‐guided triple or quadruple regimens—intention‐to‐treat eradication rates were 84.9% in the HDDT group and 78.1% in the susceptibility‐guided group; it also caused fewer adverse effects and resulted in lower overall antibiotic exposure [19]. In the present study, antibiotic susceptibility testing was performed in only a limited subset of participants, which hinders firm conclusions regarding resistance‐stratified efficacy. Because the numbers within each susceptibility category were small, observed differences are neither statistically nor clinically interpretable. Consequently, the study cannot meaningfully compare m‐HDDT with STT in clarithromycin‐resistant or hypothetical amoxicillin‐resistant populations. Future large‐scale, resistance‐stratified randomized trials should deliberately enrich for patients with clarithromycin‐ and amoxicillin‐resistant strains, then compare fexuprazan‐based m‐HDDT with standard triple therapy, bismuth‐based quadruple therapy, and susceptibility‐guided regimens. Such studies are needed to determine whether m‐HDDT can maintain high eradication rates across diverse antimicrobial resistance profiles.

Previous trials of standard triple therapy, bismuth‐based quadruple therapy, concomitant therapy, and HDDT have consistently shown that most TEAEs are gastrointestinal in nature and that overall tolerability is acceptable. In a Korean study of PPI‐based standard triple therapy, the TEAE rate was 20.4%—dyspepsia, diarrhea, abdominal pain, taste disturbance, and nausea were reported as the most frequent symptoms; the treatment discontinuation rate was only 1.8% [20]. Bismuth‐containing quadruple therapy and concomitant regimens tend to show slightly higher TEAE rates, often ranging from 20% to 30%, largely due to increased pill burden and the inclusion of metronidazole or tetracycline. These regimens are associated with more frequent diarrhea, metallic taste, nausea, and dark stools, although serious adverse events remain uncommon and most patients complete therapy [21, 22]. HDDT and vonoprazan–amoxicillin dual therapy have generally demonstrated TEAE rates similar to or lower than those of bismuth‐based quadruple or other regimens; overall adverse event incidences of approximately 15% predominantly consist of mild gastrointestinal symptoms, and discontinuation rates are very low [22, 23]. In the present study, overall incidences of TEAEs were 16.7% (16/96) in the m‐HDDT group and 14.0% (14/100) in the STT group; there was no significant difference between regimens. Additionally, no serious TEAEs were observed in either group, and no participant discontinued therapy because of adverse events, indicating a favorable safety profile for both treatments. When considered in conjunction with existing data for triple therapy, bismuth‐based quadruple therapy, concomitant therapy, and HDDT, the TEAE rate and pattern observed with fexuprazan‐based m‐HDDT fall within the expected range and do not suggest any additional tolerability burden relative to other recommended eradication regimens.

This study has several strengths. It employed a prospective, randomized, multicenter design across tertiary hospitals; included a representative Korean adult population; and incorporated parallel FAS and PP evaluations that yielded consistent results. To our knowledge, this is the first study to investigate a fexuprazan‐based high‐dose dual therapy regimen for *H. pylori* eradication. Several limitations should also be acknowledged. First, antibiotic susceptibility testing was performed in only a small subset of patients, with resistance data available for just 20 isolates. Notably, no amoxicillin‐resistant strains were identified and only a few clarithromycin‐resistant strains were detected. This limited dataset substantially restricts the interpretation of treatment efficacy in relation to antimicrobial resistance and precludes robust resistance‐stratified analyses. Consequently, the impact of antibiotic resistance on eradication outcomes could not be adequately evaluated in this study. Second, although the trial was adequately powered to demonstrate non‐inferiority for overall eradication, it was not designed to assess superiority, detect modest differences within specific subgroups, or compare alternative dosing schedules or treatment durations. Third, the dosing strategies for amoxicillin and fexuprazan used in this study may not have fully optimized pharmacodynamic exposure. In particular, the administered doses and dosing frequencies may have been insufficient to achieve maximal eradication efficacy in all patients, especially in the presence of reduced bacterial susceptibility. Therefore, the relatively modest eradication rate observed in our study may, in part, be attributable to suboptimal dosing. Further studies are warranted to explore optimized dosing regimens, including higher or more frequent administration of amoxicillin and alternative dosing strategies for fexuprazan, to improve treatment outcomes. Finally, overall eradication rates of approximately 80%—consistent with recent Korean registry data—remain below the ≥ 90% threshold increasingly proposed as a desirable benchmark. These findings indicate potential for further optimization of P‐CAB–based HDDT regimens via strategies such as extended treatment duration, refinement of amoxicillin dosing intensity, or more systematic application of susceptibility‐guided tailored therapy.

In conclusion, fexuprazan‐based m‐HDDT with bismuth achieved non‐inferior eradication rates and demonstrated comparable safety and compliance relative to PPI‐based standard triple therapy as first‐line treatment for *H. pylori* infection in Korea. Given the high and persistent prevalence of clarithromycin resistance and the global emphasis on reducing unnecessary antibiotic exposure, this clarithromycin‐sparing regimen can be an alternative therapeutic option that leverages the pharmacologic advantages of P‐CABs and bismuth. Future large‐scale, double‐blind, resistance‐stratified randomized trials comparing optimized fexuprazan‐based m‐HDDT with guideline‐recommended quadruple and tailored therapies are warranted to determine the most effective eradication strategies for *H. pylori* infection.

## Author Contributions

Jae J Kim and Ji Yong Ahn conceived and designed the study. Jae J Kim, Ji Yong Ahn, Ki‐Nam Shim, Jung‐Ho Park, Sang Gyun Kim, Jeong Hwan Kim, Jeong Seop Moon, and Young Hoon Youn contributed to patient enrollment and data acquisition. Young Hoon Youn and Jung‐Ho Park performed the statistical analysis and interpreted the data. Ji Yong Ahn drafted the manuscript. Jae J Kim and Jeong Seop Moon supervised the study and critically revised the manuscript. All authors contributed to the article and approved the submitted version.

## Funding

This study received financial support from Daewoong Pharmaceutical Co. Ltd. (Seoul, Korea).

## Conflicts of Interest

The authors declare no conflicts of interest.

## Supporting information


**Figure S1:** Randomization, interventions, assessments, and follow‐up.

**Figure S2:** The study protocol synopsis.

**Figure S3:** The statistical analysis plan.

**Table S1:** Blood chemistry laboratory parameters by treatment group.

## Data Availability

The data that support the findings of this study are available from the corresponding author upon reasonable request.
