# Supplementary figures and images for: Efficacy and Safety of Fexuprazan‐Based Modified High‐Dose Dual Therapy for Helicobacter pylori Eradication: A Randomized Clinical Trial
Source: Helicobacter. 2026 Jun 8;31(3):e70146. doi: 10.1111/hel.70146 (PMC13244396; doi:10.1111/hel.70146)

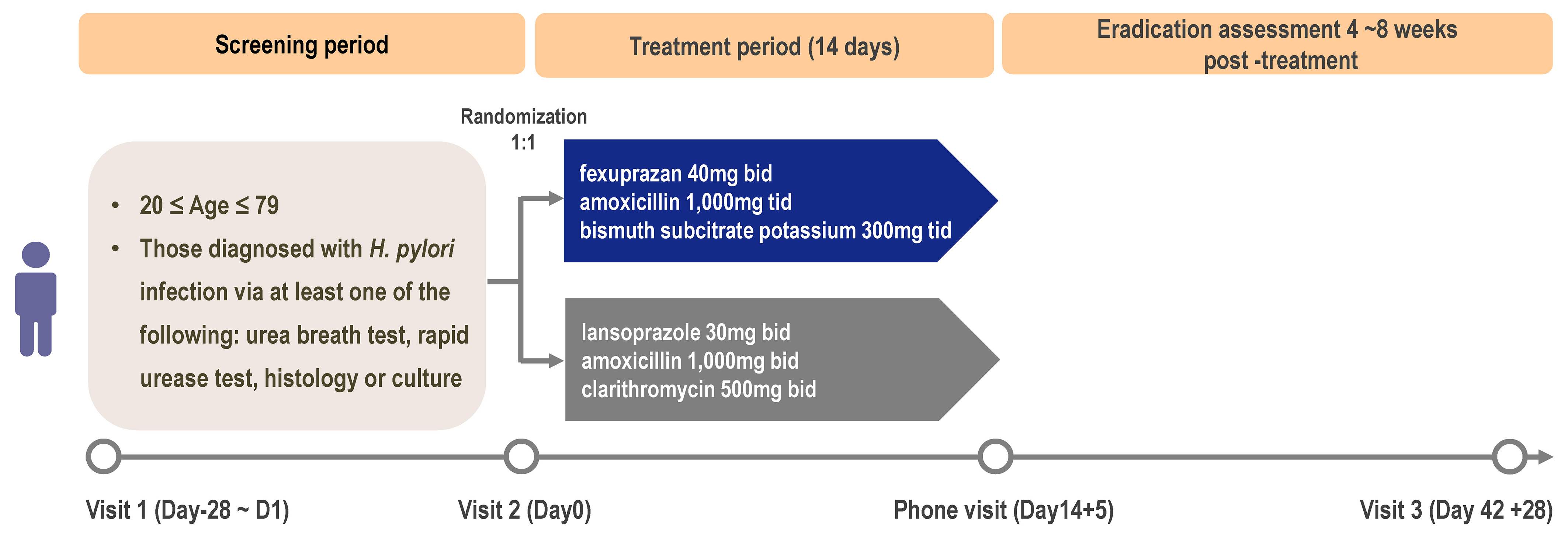

Supplement: Supplementary file 1 — Figure S1: Randomization, interventions, assessments, and follow‐up. [file HEL-31-e70146-s002.tif]
