# Supplementary material for: Efficacy and Safety of Fexuprazan‐Based Modified High‐Dose Dual Therapy for Helicobacter pylori Eradication: A Randomized Clinical Trial
Source: Helicobacter. 2026 Jun 8;31(3):e70146. doi: 10.1111/hel.70146 (PMC13244396; doi:10.1111/hel.70146)
Supplement: Supplementary file 2 — Figure S2: The study protocol synopsis. [file HEL-31-e70146-s003.pdf]

PROTOCOL SYNOPSIS

Efficacy and safety of Fexuprazan based Helicobacter pylori eradication therapy

Protocol No.	SUGR_HPE_IIT01
Version	1.1
Effective date	2023-09-26

CONFIDENTIAL

This document contains confidential information. By receiving and reviewing this document, you agree to maintain the confidentiality of the information contained herein and not to copy, disclose to any third party (except as required by applicable law or regulation), or use such information for any unauthorized purpose, unless otherwise agreed in writing.

Protocol Number	SUGR_HPE_IIT01
Study Title	Efficacy and safety of Fexuprazan based Helicobacter pylori eradication therapy
Principle Investigator	Ji Yong Ahn, Division of Gastroenterology, Department of Internal Medicine, University of Ulsan College of Medicine, Asan Medical Center
Study Centers	Asan Medical Center and 7 additional sites in Korea
Study Period	▪ Total study period: 24 months from IRB approval<br>▪ Study period for each subject: approximately 10–14 weeks (Screening period up to 4 weeks + treatment period 2 weeks + evaluation period approximately 4–8 weeks)
Target Disease	Patients confirmed to be positive for Helicobacter pylori infection
Objective	To compare the Helicobacter pylori eradication efficacy of fexuprazan-based triple therapy (fexuprazan 40 mg twice daily, amoxicillin 1000 mg three times daily, and potassium bismuth citrate 300 mg three times daily) administered orally for 14 days with that of PPI-based standard triple therapy (lansoprazole 30 mg, amoxicillin 1000 mg, and clarithromycin 500 mg twice daily) administered for 14 days in patients positive for Helicobacter pylori infection.
Number of Subjects	Planned number of subjects: total of 180 subjects<br>(Minimum number of subjects per group: 81; total 90 subjects per group considering a 10% dropout rate)
Study Design	Multicenter, prospective, randomized, open-label, active-controlled, investigator-initiated clinical trial
Inclusion Criteria	1. Male or female subjects aged 20 years or older and less than 80 years<br>2. Subjects diagnosed as Helicobacter pylori positive by at least one of the following tests: urea breath test, rapid urease test, or histologic examination<br>3. Subjects with atrophic gastritis, peptic ulcer disease, low-grade MALT lymphoma, functional dyspepsia, idiopathic thrombocytopenic purpura, iron deficiency anemia, hyperplastic

	polyps, history of endoscopic resection for gastric cancer or gastric adenoma, or family history of gastric cancer<br>4. Subjects who voluntarily agreed to participate in this study and signed the written informed consent form
Exclusion Criteria	1. Subjects who had previously received Helicobacter pylori eradication therapy before participation in the study<br>2. Subjects with a history of hypersensitivity to the investigational product or the active ingredients/excipients of penicillin-class antibiotics or macrolide-class antibiotics<br>3. Subjects taking contraindicated concomitant medications for the investigational products<br>4. Subjects who had taken proton pump inhibitors, histamine receptor antagonists, or potassium-competitive acid blockers within 2 weeks before initiation of study treatment, or who required medications other than study drugs including proton pump inhibitors, histamine receptor antagonists, or potassium-competitive acid blockers during the study period after randomization<br>5. Subjects who had taken antibiotics or bismuth within 4 weeks before upper gastrointestinal endoscopy and urea breath test, or who required antibiotics or bismuth within 4 weeks before randomization and during the study period after randomization<br>6. Subjects with renal or hepatic impairment who showed the following abnormal laboratory findings in blood chemistry tests<br>- AST, ALT, ALP, γ-GT, or total bilirubin levels ≥2 times the upper normal limit (UNL) at each study site<br>- BUN or creatinine levels ≥1.5 times the upper normal limit (UNL) at each study site<br>7. Subjects with central nervous system infection or electrolyte abnormalities (hypokalemia, hypomagnesemia)<br>8. Subjects showing abnormal ECG findings including QT interval prolongation on screening electrocardiogram<br>9. Subjects with gastrointestinal bleeding on upper gastrointestinal endoscopy

	10. Subjects with uncontrolled diabetes mellitus, hypertension, or abnormal liver function<br>11. Subjects with a history of gastrointestinal malignancy within 5 years<br>12. Subjects with a history of esophageal or gastric resection<br>13. Subjects with hereditary problems such as galactose intolerance, Lapp lactase deficiency, or glucose-galactose malabsorption<br>14. Alcoholics<br>15. Subjects who did not agree to use appropriate contraception during the clinical trial period<br>16. Pregnant or breastfeeding women<br>17. Subjects judged by the investigator to be inappropriate for participation in the clinical trial
Study Procedure	In this study, a total of three scheduled visits, including the screening period (Visit 1), randomization visit (Visit 2), and follow-up visit (Visit 3), as well as one telephone visit (D14+5), are planned. Visit 1 and Visit 2 may be performed on the same day, and examinations and procedures performed at Visit 1 will not be repeated at Visit 2 (however, the examinations and procedures at Visit 1 must be completed before the final confirmation of the inclusion/exclusion criteria).<br><br>If necessary during the course of the study, subjects may have additional unscheduled visits (USV) separate from the visits planned in the clinical trial protocol. For example, if the occurrence of an adverse event is suspected, or for follow-up after the occurrence of an adverse event, the subject may visit the study site and undergo necessary examinations, and the investigator shall inform the subject of such circumstances.<br><br>All visits related to the clinical trial shall be documented in both the source documents and the case report form. However, unscheduled

	visits shall not result in changes to the schedule specified in the clinical trial protocol.
Randomization Method	The randomization code for this clinical trial will be generated by a statistician who is not directly involved in this clinical trial using SAS® 9.4 64bit (SAS Institute Inc., Cary, NC, USA) or a later version, such that subjects are allocated to each treatment group at a 1:1 ratio according to the block randomization method by study site.<br><br>This study is an open-label study, and subjects who meet the inclusion/exclusion criteria will be assigned sequentially to each treatment group according to the randomization code distributed to each study site.<br><br>Thereafter, subjects who meet the inclusion/exclusion criteria will be assigned sequentially to each treatment group according to the randomization code using the interactive web response system (IWRS).
Discontinuation and Dropout Criteria	1. Withdrawal of consent by the subject<br>2. Use of medications that may affect study results without the judgment of the principal investigator or study personnel during the clinical trial period<br>3. Participation of subjects who do not meet the inclusion/exclusion criteria<br>4. Requirement for additional treatment or procedures due to worsening of disease<br>5.Discovery of clinically significant systemic disease not identified during pre-treatment examinations<br>6. Inability to follow up because the subject did not attend scheduled visits<br>7.Cases in which the investigator judges that continuation of the clinical trial is difficult due to safety issues such as adverse events

	8. Cases in which the subject requests discontinuation of investigational product administration due to safety issues such as adverse events<br>9. Pregnancy or confirmed pregnancy during the clinical trial period (from the date of informed consent to study discontinuation or until 4 weeks after completion of investigational product administration)<br>10. Cases in which the principal investigator or study personnel determine that continuation of the clinical trial is no longer possible
Investigational Products and Treatment Method	▪ Test group: Fexuprazan 40 mg BID + Amoxicillin 1000 mg TID + Potassium bismuth citrate 300 mg TID for 14 days<br>▪ Control group: Lansoprazole 30 mg BID + Amoxicillin 1000 mg BID + Clarithromycin 500 mg BID for 14 days
Efficacy Endpoints	▪ Primary efficacy endpoint:<br>Eradication rate at 4 to 8 weeks after completion of medication<br><br>▪ Secondary efficacy endpoints:<br>1. Eradication rate according to antibiotic resistance of Helicobacter pylori strains<br>2. Drug compliance
Safety Endpoints	Adverse events, Vital signs, Blood chemistry tests, Physical examination

Schedule of Assessments

	Screening	Treatment period		f/up period
Visit number	Visit 1[13)]	Visit 2[13)]	Telephone Visit	Visit 3
Day	Day -28 ~	Day 0	Day 14+5	Day 42+28
Informed Consent	O			
Demographics [1)]	O			
Medical History[2)]	O			
Physical Examination	O	O		O
Height, Weight[3)]	O	O		O
Vital Signs[4)]	O	O		O
Laboratory Tests[5)]	O			O
Pregnancy Test[6)]	O			O
ECG[7)]	O			
GI Symptom Assessment[8)]	O	O		
Upper GI Endoscopy [9)]	O			
H. pylori Antibiotic Resistance Test10[10)]	O			
H. pylori Test [11)]	O			
Urea Breath Test				O[14)]
Inclusion/Exclusion Criteria	O	O		
Randomization		O		

Prescription of Investigational Product		O		
Prior/Concomitant Medication 12)	O	O		O
Dispensing of Investigational Product		O		
Compliance Assessment			O	O
Adverse Event Assessment			O	O

1) Demographic information: initials, sex, date of birth, age, smoking history, and alcohol consumption history will be collected.
2) Medical history: medical history within 6 months or ongoing medical history as of Visit 1 (screening visit) will be collected.
3) Height will be measured only at screening.
4) Vital signs: body temperature, sitting blood pressure, and pulse rate (measured after 5 minutes of rest) will be assessed.
5) Test results obtained within 4 weeks (28 days) before Visit 1 (screening visit) may be used. Screening tests must be performed and confirmed before Visit 2 (randomization).
6) Pregnancy testing will be performed in all subjects with childbearing potential using urine HCG testing (serum HCG testing may be used if urine HCG testing is not feasible).
7) ECG results obtained within 4 weeks (28 days) before Visit 1 (screening visit) may be used.
8) Gastrointestinal symptom assessment will evaluate the presence of symptoms including abdominal pain, nausea/vomiting, heartburn, chest burning, acid reflux, dyspepsia, and others.
9) Histologic examination will be performed when suspicious lesions requiring biopsy are identified.
10) If Helicobacter pylori infection is confirmed by urea breath test before endoscopy, one specimen each from the gastric antrum and body will be collected during upper GI endoscopy for antibiotic resistance testing.

11) Subjects positive for Helicobacter pylori by at least one of the following within 4 weeks (28 days) before Visit 1 are eligible: urea breath test, rapid urease test, or histologic examination.
12) Medications taken within 4 weeks (28 days) before Visit 1 (screening visit) will be investigated.
13) Visit 1 (screening visit) and Visit 2 (randomization visit) may be performed on the same day if all inclusion and exclusion criteria are confirmed.
14) In cases of eradication treatment failure, second-line bismuth quadruple therapy (bismuth + metronidazole + tetracycline + proton pump inhibitor for 2 weeks) will be administered according to the 2020 Korean guidelines.
