# Supplementary material for: Efficacy and Safety of Fexuprazan‐Based Modified High‐Dose Dual Therapy for Helicobacter pylori Eradication: A Randomized Clinical Trial
Source: Helicobacter. 2026 Jun 8;31(3):e70146. doi: 10.1111/hel.70146 (PMC13244396; doi:10.1111/hel.70146)
Supplement: Supplementary file 3 — Figure S3: The statistical analysis plan. [file HEL-31-e70146-s004.pdf]

STATISTICAL ANALYSIS PLAN

Efficacy and safety of Fexuprazan based Helicobacter pylori eradication therapy

Protocol No.	SUGR_HPE_IIT01
Version	1.1
Effective date	2023-09-26

CONFIDENTIAL

This document contains confidential information. By receiving and reviewing this document, you agree to maintain the confidentiality of the information contained herein and not to copy, disclose to any third party (except as required by applicable law or regulation), or use such information for any unauthorized purpose, unless otherwise agreed in writing.

Protocol Number

SUGR_HPE_IIT01

Study Title

Efficacy and safety of Fexuprazan based Helicobacter pylori eradication therapy

Sponsor-Investigator

Prof. Ji Yong Ahn, Department of Gastroenterology, Asan Medical Center

Study Centers and Principal Investigators

Asan Medical Center and 7 additional sites in Korea

Table of contents

1. Objective 4

2. Target Disease 4

3. Study Period 4

4. Number of Subjects 4

5. Study Design 5

6. Study Procedure 5

7. Randomization Method 6

8. Investigational Products and Treatment Method 6

9. Efficacy Endpoints 7

10. Safety Endpoints 7

11. Statistical Analysis 7

1) Sample size and analysis populations 7

2) Randomisation and treatment assignment 8

3) Baseline characteristics 8

4) Primary efficacy analysis 9

5) Secondary efficacy analyses 9

6) Safety analysis 10

7) Handling of missing data and software 11

1. Objective

To compare the Helicobacter pylori eradication efficacy of fexuprazan-based triple therapy (fexuprazan 40 mg twice daily, amoxicillin 1000 mg three times daily, and potassium bismuth citrate 300 mg three times daily) administered orally for 14 days with that of PPI-based standard triple therapy (lansoprazole 30 mg, amoxicillin 1000 mg, and clarithromycin 500 mg twice daily) administered for 14 days in patients positive for Helicobacter pylori infection.

2. Target Disease

Patients confirmed to be positive for Helicobacter pylori infection

3. Study Period

▪ Total study period: 24 months from IRB approval

▪ Study period for each subject: approximately 10–14 weeks

(Screening period up to 4 weeks + treatment period 2 weeks + evaluation period approximately 4–8 weeks)

4. Number of Subjects

Planned number of subjects: total of 180 subjects

(Minimum number of subjects per group: 81; total 90 subjects per group considering a 10% dropout rate)

5. Study Design

Multicenter, prospective, randomized, open-label, active-controlled, investigator-initiated clinical trial

6. Study Procedure

This study consists of three scheduled visits including the screening period (Visit 1), randomization visit (Visit 2), and follow-up visit (Visit 3), as well as one telephone visit (D14+5).

Visit 1 and Visit 2 may be performed on the same day, and examinations and procedures performed at Visit 1 will not be repeated at Visit 2.

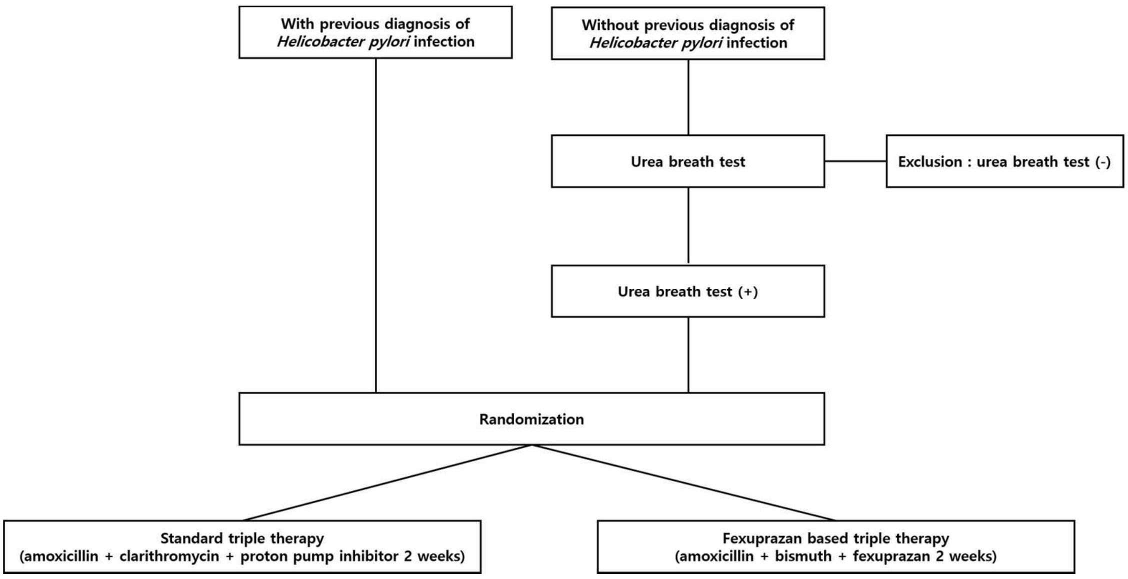

7. Randomization Method

The randomization code for this clinical trial will be generated by a statistician who is not directly involved in this clinical trial using SAS® 9.4 64bit (SAS Institute Inc., Cary, NC, USA) or a later version, such that subjects are allocated to each treatment group at a 1:1 ratio according to the block randomization method by study site.

This study is an open-label study, and subjects who meet the inclusion/exclusion criteria will be assigned sequentially to each treatment group according to the randomization code distributed to each study site.

Thereafter, subjects who meet the inclusion/exclusion criteria will be assigned sequentially to each treatment group according to the randomization code using the interactive web response system (IWRS).

8. Investigational Products and Treatment Method

Group	Investigational Product	Administration	Treatment period
Test	Fexuprazan 40 mg	BID	14 days
	Amoxicillin 1000 mg	TID	
	Potassium bismuth citrate 300 mg	TID	
Control	Lansoprazole 30 mg<br>Amoxicillin 1000 mg<br>Clarithromycin 500 mg	BID	14 days

9. Efficacy Endpoints

▪ Primary efficacy endpoint:

Eradication rate at 4 to 8 weeks after completion of medication

▪ Secondary efficacy endpoints:

- Eradication rate according to antibiotic resistance of Helicobacter pylori strains
- Drug compliance

10. Safety Endpoints

- Adverse events
- Vital signs
- Blood chemistry tests
- Physical examination

11. Statistical Analysis

1) Sample size and analysis populations

The trial was designed as a non-inferiority comparison of a Fexuprazan-based modified HDDT regimen against a Lansoprazole-based standard triple therapy regimen for *Helicobacter pylori* eradication. The pre-specified eradication rates were 83% in the test arm (informed by the vonoprazan dual-therapy report by Chey *et al.* and the bismuth-augmentation benefit reported by Kim *et al.*) and 75% in the control arm (Kim *et al.*, 2011). With a non-inferiority margin of −10% on the absolute risk difference (test minus control), a one-sided significance level of 2.5%, a power of 80%, and 1:1 allocation, 81 evaluable subjects per arm were required, computed with the “Non-Inferiority Tests for the Difference Between Two Proportions” procedure in PASS 2020 (NCSS, LLC,

Kaysville, UT, USA). Allowing for a 10% drop-out rate, 90 subjects per arm (180 in total) were planned for enrolment. A total of 196 subjects were randomised, 96 to the test (Fexuprazan-based modified HDDT) group and 100 to the control (standard triple therapy) group.

Three analysis populations were defined. The **Full Analysis Set (FAS)** comprised all randomised subjects who were *H. pylori*-positive at screening and received at least one dose of the investigational product, and was the primary analysis set for efficacy. The **Per-Protocol Set (PP)** comprised FAS subjects who completed treatment without major protocol violations and with overall study-medication adherence ≥ 80%, and was the supportive analysis set for efficacy. The **Safety Set (SS)** comprised all randomised subjects who received at least one dose of the investigational product, and was the analysis set for safety.

2) Randomisation and treatment assignment

Eligible subjects were randomised 1:1 to the test or control regimen using site-stratified block randomisation. The randomisation codes were generated by an independent statistician with SAS® version 9.4 (SAS Institute Inc., Cary, NC, USA) and were administered through an Interactive Web Response System (IWRS) in the order of enrolment. The trial was open-label.

3) Baseline characteristics

Demographic and baseline characteristics were summarised on the FAS by treatment group. Continuous variables (age, BMI) were summarised as mean ± SD, median, minimum and maximum, and categorical variables (gender, smoking, alcohol, gastrointestinal symptom assessment, indications for *H. pylori* eradication, history of eradication therapy, history of gastrointestinal disease, medical history, and allergy status) as n (%). Between-group comparisons used the independent two-sample t-test or

the Wilcoxon rank-sum test for continuous variables, and the χ^2 or Fisher's exact test for categorical variables. Fisher's exact test was used in place of χ^2 when expected cell counts were below 5 in more than 20% of cells.

4) Primary efficacy analysis

The primary efficacy endpoint was the *H. pylori* eradication rate at 4–8 weeks after the end of treatment, assessed by ^{13}C-urea breath test at Visit 3. Eradication success was defined as a negative urea breath test obtained between 28 and 56 days after the end of treatment (Visit 2 + 42 days ≤ Visit 3 ≤ Visit 2 + 70 days). A positive urea breath test, an assessment outside the 28–56-day window, or a missed Visit 3 was considered eradication failure.

Eradication rates with two-sided 95% Wald confidence intervals (CIs) were summarised by treatment group on the FAS and the PP set. The between-group rate difference (test − control) was reported with its two-sided 95% Wald CI, together with a one-sided Wald non-inferiority p-value computed using the unpooled-variance Z statistic. With a pre-specified non-inferiority margin of −10%, non-inferiority was concluded when the lower bound of the 95% CI for the rate difference exceeded −10%.

5) Secondary efficacy analyses

The following secondary efficacy analyses were performed on the FAS. **(1) Eradication rate by antibiotic resistance.** In the subset of subjects with available *H. pylori* culture and susceptibility results, the eradication rate, the two-sided 95% Wald CI, and the between-group p-value (Fisher's exact test, with χ^2 where appropriate) were presented separately for amoxicillin-resistant or -susceptible subgroups, for clarithromycin-resistant or -susceptible subgroups, and for the combined amoxicillin-and-clarithromycin resistance status (resistant to either antibiotic vs. susceptible to both). **(2) Drug adherence.** Adherence was calculated as (actual amount taken ÷ amount prescribed) × 100 and was

summarised both as a continuous variable (mean ± SD, median, minimum, maximum) and as a binary variable (≥ 80% vs. < 80%) by treatment group. The continuous comparison used the Wilcoxon rank-sum test, and the binary comparison used Fisher's exact test. **(3) Eradication rate by drug-adherence category.** Eradication rates stratified by adherence category (< 80% vs. ≥ 80%) were compared between treatment groups using χ^2 or Fisher's exact tests. **(4) Eradication after second-line rescue therapy.** Among subjects who proceeded to second-line bismuth quadruple rescue therapy, the eradication rate, 95% Wald CI, risk difference, a one-sided Wald non-inferiority p-value, and a Fisher's exact p-value were reported by treatment group. **(5) Upper-GI endoscopy findings at screening.** Treatment-group comparisons of oesophageal, gastric, and duodenal endoscopy findings at screening were performed using χ^2 or Fisher's exact tests.

6) Safety analysis

All safety analyses were performed on the SS by treatment group. Adverse events were coded with the Medical Dictionary for Regulatory Activities (MedDRA). A treatment-emergent adverse event (TEAE) was defined as any adverse event that first occurred after the first dose of the investigational product, or a pre-existing adverse event that worsened after the first dose. The number of subjects with at least one TEAE, the number of TEAEs, and the corresponding percentages were summarised by treatment group; the most common TEAEs (preferred term, ≥ 2% in any group) were tabulated. Between-group comparisons of the overall TEAE rate were performed using the χ^2 test, with Fisher's exact test substituted when expected cell counts were below 5 in more than 20% of cells. Serious adverse events, adverse events leading to discontinuation, and adverse events leading to death were summarised in subject-level listings.

Blood-chemistry parameters (total protein, albumin, total bilirubin, AST, ALT, γ-GT, BUN, creatinine, alkaline phosphatase, glucose, total cholesterol, triglyceride) were summarised at baseline and at Visit 3 by treatment group; within-group changes from

baseline were tested with the paired t-test, and between-group differences in change from baseline with the independent two-sample t-test. Haematology, electrolyte panel, vital signs, and physical-examination findings are presented as subject-level listings.

7) Handling of missing data and software

For the primary efficacy endpoint, subjects with no valid post-treatment urea breath test (because of a missed Visit 3 or an assessment outside the 28–56-day window) and subjects who discontinued early were considered eradication failures. For safety analyses, missing values were treated as missing without imputation.

All statistical tests were two-sided at the 5% significance level unless otherwise specified; the non-inferiority test was one-sided at the 2.5% significance level. P-values were rounded to four decimal places, percentages to one decimal place, and continuous variables to two decimal places. Analyses were performed with SAS® version 9.4 (SAS Institute Inc., Cary, NC, USA), and figures were prepared with R version 4.x or higher (R Foundation for Statistical Computing, Vienna, Austria).
