# Supplementary material for: Efficacy and Safety of Fexuprazan‐Based Modified High‐Dose Dual Therapy for Helicobacter pylori Eradication: A Randomized Clinical Trial
Source: Helicobacter. 2026 Jun 8;31(3):e70146. doi: 10.1111/hel.70146 (PMC13244396; doi:10.1111/hel.70146)
Supplement: Supplementary file 4 — Table S1: Blood chemistry laboratory parameters by treatment group. [file HEL-31-e70146-s001.docx]

Supplementary Table 1. Blood chemistry laboratory parameters by treatment group

|  | |  | | | Observed value | | | Change from baseline | | | | | |
| --- | --- | --- | --- | --- | --- | --- | --- | --- | --- | --- | --- | --- | --- |
| Parameter | Treatment | | Visit | n | | Mean | SD | | *p* value | n | Mean | SD | *p* value |
| Albumin | m-HDDT group | | Baseline | 96 | | 4.45 | 0.29 | |  |  |  |  |  |
|  |  | | Visit 3 | 92 | | 4.47 | 0.30 | | 0.7464 | 92 | 0.01 | 0.22 | 0.4633 |
|  | STT group | | Baseline | 100 | | 4.42 | 0.30 | |  |  |  |  |  |
|  |  | | Visit 3 | 98 | | 4.40 | 0.29 | | 0.4611 | 98 | -0.02 | 0.20 |  |
| Alkaline phosphatase | m-HDDT group | | Baseline | 96 | | 76.78 | 29.77 | |  |  |  |  |  |
|  |  | | Visit 3 | 92 | | 77.76 | 31.18 | | 0.8373 | 92 | 0.18 | 8.61 | 0.8155 |
|  | STT group | | Baseline | 100 | | 80.15 | 51.52 | |  |  |  |  |  |
|  |  | | Visit 3 | 98 | | 81.04 | 50.05 | | 0.7191 | 98 | 0.66 | 18.21 |  |
| ALT | m-HDDT group | | Baseline | 96 | | 22.57 | 10.90 | |  |  |  |  |  |
|  |  | | Visit 3 | 92 | | 25.28 | 15.73 | | 0.0655 | 92 | 2.57 | 13.20 | 0.4512 |
|  | STT group | | Baseline | 100 | | 20.94 | 10.36 | |  |  |  |  |  |
|  |  | | Visit 3 | 98 | | 22.01 | 12.36 | | 0.1967 | 98 | 1.29 | 9.79 |  |
| AST | m-HDDT group | | Baseline | 96 | | 24.36 | 6.04 | |  |  |  |  |  |
|  |  | | Visit 3 | 92 | | 26.53 | 8.75 | | 0.0138 | 92 | 2.23 | 8.51 | 0.2993 |
|  | STT group | | Baseline | 100 | | 24.48 | 7.31 | |  |  |  |  |  |
|  |  | | Visit 3 | 98 | | 25.53 | 7.98 | | 0.2049 | 98 | 1.00 | 7.76 |  |
| BUN | m-HDDT group | | Baseline | 96 | | 14.47 | 3.45 | |  |  |  |  |  |
|  |  | | Visit 3 | 92 | | 14.74 | 4.01 | | 0.4901 | 92 | 0.23 | 3.22 | 0.5274 |
|  | STT group | | Baseline | 100 | | 15.23 | 4.58 | |  |  |  |  |  |
|  |  | | Visit 3 | 98 | | 15.88 | 4.46 | | 0.1788 | 98 | 0.58 | 4.21 |  |
| Creatinine | m-HDDT group | | Baseline | 96 | | 0.83 | 0.19 | |  |  |  |  |  |
|  |  | | Visit 3 | 92 | | 0.82 | 0.18 | | 0.0087 | 92 | -0.02 | 0.08 | 0.2915 |
|  | STT group | | Baseline | 100 | | 0.84 | 0.20 | |  |  |  |  |  |
|  |  | | Visit 3 | 98 | | 0.83 | 0.19 | | 0.317 | 98 | -0.01 | 0.09 |  |
| Glucose | m-HDDT group | | Baseline | 96 | | 104.18 | 17.45 | |  |  |  |  |  |
|  |  | | Visit 3 | 92 | | 103.58 | 14.85 | | 0.5284 | 92 | -0.78 | 11.86 | 0.2107 |
|  | STT group | | Baseline | 100 | | 105.35 | 19.81 | |  |  |  |  |  |
|  |  | | Visit 3 | 98 | | 107.07 | 18.43 | | 0.2628 | 98 | 1.56 | 13.72 |  |
| Total bilirubin | m-HDDT group | | Baseline | 96 | | 0.78 | 0.40 | |  |  |  |  |  |
|  |  | | Visit 3 | 92 | | 0.76 | 0.32 | | 0.7923 | 92 | -0.01 | 0.24 | 0.2835 |
|  | STT group | | Baseline | 100 | | 0.71 | 0.35 | |  |  |  |  |  |
|  |  | | Visit 3 | 98 | | 0.76 | 0.44 | | 0.2683 | 98 | 0.04 | 0.37 |  |
| Total cholesterol | m-HDDT group | | Baseline | 96 | | 183.95 | 41.82 | |  |  |  |  |  |
|  |  | | Visit 3 | 92 | | 183.35 | 42.35 | | 0.878 | 92 | -0.42 | 26.41 | 0.3645 |
|  | STT group | | Baseline | 100 | | 184.79 | 48.42 | |  |  |  |  |  |
|  |  | | Visit 3 | 98 | | 180.12 | 42.92 | | 0.1769 | 98 | -4.20 | 30.60 |  |
| Total protein | m-HDDT group | | Baseline | 96 | | 7.22 | 0.48 | |  |  |  |  |  |
|  |  | | Visit 3 | 92 | | 7.20 | 0.40 | | 0.4915 | 92 | -0.02 | 0.32 | 0.9242 |
|  | STT group | | Baseline | 100 | | 7.22 | 0.44 | |  |  |  |  |  |
|  |  | | Visit 3 | 98 | | 7.20 | 0.36 | | 0.5801 | 98 | -0.02 | 0.33 |  |
| Triglycerides | m-HDDT group | | Baseline | 96 | | 139.88 | 106.14 | |  |  |  |  |  |
|  |  | | Visit 3 | 92 | | 131.46 | 81.21 | | 0.3172 | 92 | -9.24 | 88.11 | 0.5236 |
|  | STT group | | Baseline | 100 | | 134.46 | 84.61 | |  |  |  |  |  |
|  |  | | Visit 3 | 98 | | 116.32 | 58.84 | | 0.0078 | 98 | -16.24 | 59.19 |  |
| γ-GT | m-HDDT group | | Baseline | 96 | | 25.80 | 17.07 | |  |  |  |  |  |
|  |  | | Visit 3 | 92 | | 30.95 | 26.81 | | 0.0105 | 92 | 4.66 | 17.11 | 0.7998 |
|  | STT group | | Baseline | 100 | | 27.02 | 23.09 | |  |  |  |  |  |
|  |  | | Visit 3 | 98 | | 30.68 | 36.93 | | 0.2761 | 98 | 3.69 | 33.39 |  |

m-HDDT group, fexuprazan-based modified high-dose dual therapy group; STT group, proton pump inhibitor–based standard triple therapy group; AST, aspartate aminotransferase; ALT, alanine transaminase; BUN, blood urea nitrogen; γ-GT, gamma-glutamyl transferase

*P* values were calculated using paired t-tests for within-group changes from baseline and independent two-sample t-tests for between-group differences in changes from baseline.
